# Complementary and alternative medicine use among children with mental health issues: results from the National Health Interview Survey

**DOI:** 10.1186/s12906-018-2307-5

**Published:** 2018-08-29

**Authors:** Claudia Wang, John Preisser, Yunro Chung, Kaigang Li

**Affiliations:** 1 School of Physical Education, Pingdingshan University, South Weilai Rd, Pingdingshan City New District, Pingdingshan, Henan Province 467000 People’s Republic of China; 2 Department of Biostatistics, the University of North Carolina at Chapel Hill, Chapel Hill, NC 27516 USA; 3 Fred Hutchinson Cancer Research, Seattle, WA 98109 USA; 4 Department of Health & Exercise Science, Colorado State University, Fort Collins, CO 80523 USA

**Keywords:** Complementary and alternative medicine, Mental health, Children

## Abstract

**Background:**

Mental health issues in children have become a serious public health concern in the U.S. within the past few decades. Emerging evidence suggests that Complementary and Alternative Medicine (CAM) can be beneficial for various mental health issues. However, little is known about the prevalence, reasons, and associated factors of CAM use among this population in the U.S. The purpose of this study is to examine the characteristics of CAM use among U.S. children with mental health issues.

**Methods:**

Utilizing the 2012 National Health Interview Survey data, we used descriptive analysis, Wald F-statistics, and multivariable survey logistic regression models to examine the prevalence, patterns, and associated factors of CAM use in children aged 4–17 (*n* = 10,233) adjusting for the complex sampling design.

**Results:**

CAM use is more popular among children with mental health issues compared with those without (19.2% vs. 10.1%, *p* < 0.001). Herbal remedies (9.1%), mind-body therapies (5.5%), and chiropractic care (5.3%) were the most frequently used modalities. Primary reasons for children to use CAM are because they are helpful (69.2%), natural (55.9%), and holistic (44.7%). The majority of CAM users perceived CAM therapies are helpful. Predictors of CAM use are children who are female, whose parents had a higher educational level and socioeconomic status, and who had at least one co-morbid medical condition. Only 18.4% of CAM usage was recommended by medical doctors.

**Conclusions:**

Approximately 10 million parents of children with mental health issues reported the use of CAM therapies, mainly because of their desire for a more natural and holistic healthcare approach. Given that the majority of CAM users perceived CAM therapies as helpful, future studies should investigate the unique contributions of CAM in pediatric psychiatric care. Because a low percentage of CAM use was recommended by medical doctors, educational interventions designed to equip medical professionals with CAM knowledge and experience will be conducive to improving effective patient-physician communication in clinical settings. Since CAM use is reported as more prevalent by parents’ of children with higher education and family income, effective strategies designed to reduce disparities in accessing promising CAM therapies are warranted.

## Background

Mental health issues (e.g., attention-deficit hyperactivity disorder (ADHD), anxiety/depression, chronic stress, and autism) in children have become a serious public health concern in the United States especially within the past few decades. According to the data from National Health Interview Survey (NHIS), the prevalence of ADHD in children and adolescents rose from 5.9% in 1998 to 8.2% in 2007 and 11.4% in 2011 [1–3]. Also, the proportion of teens with depression disorders increased from 26.1% in 2009 to 29.9% in 2015 [4–6]. Moreover, approximately one in five children are experiencing one or more types of mental disorders, which has resulted in a cost of $247 billion each year according to the 2013 report from the Mental Health Surveillance among Children in the United States (U.S.) [7].

Mental health disorders that are not well treated can lead to other more serious health problems, which could in turn adversely affect children’s family/peer relationships, academic performance, and social functioning [8–10]. Moreover, children with mental health issues are more likely to encounter other medical comorbidities, such as asthma, respiratory allergies, and sleep difficulties [11–16]. Furthermore, childhood mental health problems often continue into adulthood and lead to decreased productivity, increased substance abuse, and substantial economic burden to the individual and society [17, 18].

For decades, the most commonly used conventional medical treatments for mental health issues in children were pharmaceutical medications and psychological counseling [19–21]. Although these conventional approaches continue as the dominant medical model, recently many parents who have children with mental health issues have been reluctant to use these conventional methods because of their concern about the side effects, being stigmatized as failures, withdrawal symptoms, life-long reliance, and/or difficulty affording mental health counseling [22–24]. Instead, many parents have turned their children to a variety of complementary and alternative medicine (CAM) therapies (e.g., natural products, manipulative/body-based techniques, and mindfulness-based practices, etc.,), with the desire for a more natural and holistic treatment as well as the hope to minimize the risk of adverse effects caused by pharmaceutical medications [3, 25, 26].

The appeal of these non-conventional therapies is understandable. Emerging evidence indicates that CAM therapies may help children to reduce symptoms for a wide range of mental health issues, including ADHD/ADD, autism, anxiety, depression, and stress [23, 27, 28]. For instance, many studies have suggested mindfulness-based practices (e.g., yoga, tai chi, qigong, and meditation) may be a beneficial adjunct to the treatment of mental health problems, particularly mood and anxiety disorders [29–32]. Evidence also suggest that herbal supplements and natural products (e.g., St. John’s Wort, *Ginkgo biloba*, Ginseng, and Lemon Balm) can serve as promising therapeutics for childhood stress, anxiety, and depression, and help children with ADHD in reducing the difficulties of concentration and hyperactivity [24, 33, 34]. Moreover, some recent systematic reviews and meta-analyses have also demonstrated CAM therapies can reduce mental health symptoms, such as anxiety, depression, and chronic stress, and improving quality of life [35–38].

The promising profile of CAM therapies, the natural and holistic philosophies, and the availability of CAM products and practices, coincide an increasing trend of CAM use in the U.S. Indeed, several studies have shown that nearly half of Americans use CAM—often in conjunction with conventional care, and this trend is likely to continue [1, 39–41]. However, few studies have specifically examined the prevalence of CAM use among children with mental health issues at the national level. The paucity of studies specially exploring CAM use among children with mental health issues has also indicated that there is little knowledge of the reasons, patterns, and associated factors with CAM use. Addressing this gap may assist in further understanding of the needs of parents who have children with mental health issues and the factors influencing their CAM use behaviors. Insights gained from such research may also be valuable in informing CAM practice, education, policy, as well as future directions of CAM research for this specific population.

For these reasons, the purposes of our study are to 1) describe the prevalence of CAM use and identify the most frequently used CAM therapies, 2) examine the patterns of and reasons for using CAM, and 3) to explore the associated factors of CAM use among children with mental health issues utilizing the most updated NHIS Child Complementary and Alternative Medicine (CAL) supplement data.

## Methods

### Data sources

We examined data from the 2012 National Health Interview Survey (NHIS), which is conducted by the National Center for Health Statistics, Centers for Disease Control and Prevention [42]. NHIS gathers data on the health of the civilian, non-institutionalized population in the U.S. by randomly selecting households with a multistage stratified design. Generally, NHIS collects data using four main components: the household, family, sample child, and sample adult core surveys. In 2012, the NCHS added the CAL supplement to the regular NHIS of previous years to gather extensive information about CAM use by U.S. children nationwide.

Data used in this study specifically came from the Family Core, Sample Child Core, and the CAL supplement of 2012 NHIS. A sample child (SC) in each family was randomly selected for the Sample Child Core and CAL supplement. The Family Core collected data on socio-demographics, insurance status, and utilization of health care services for each family member. The Sample Child Core collected data on children’s medical conditions, prescription medication use, and use of and access to traditional medical care. A knowledgeable adult family member in the household responded to the questionnaires regarding the child’s health. The CAL supplement asked adult respondents about the sample child’s use of CAM therapies within the 12 months prior to the survey year. Specifically, the 2012 CAL supplement added a series of questions, such as the reasons for using these CAM therapies, their perceived helpfulness in treating specific health issues, the information sources of the CAM therapies, and referrals for them to use these CAM therapies. Thus, it has created an opportunity for researchers to have a better understanding of CAM use among children with specific health issues [1, 43].

### Measures

#### Mental health issues

For this study, children were classified as having mental health issues if their family members answered “yes” to one of the following five questions: 1) *Has a doctor or health professional ever told you that [sample child] had ADHD or ADD?* 2) *Has a doctor or other health professional told you that [sample child] had autism?* 3) *Has a doctor or other health professional told you that [sample child] had depression?* 4) *Has a doctor or other health professional told you that [sample child] frequently felt anxious, nervous, or worried (anxiety)?* 5) *Has a doctor or other health professional told you that [sample child] frequently felt stressed?* Based on previous studies and the frequencies from the 2012 NHIS report, we combined feeling anxious, nervous, worried, and depression into one category of anxiety/depression for analyses, due to the small sample size and similar symptoms/categories of mental health conditions [2].

#### CAM use

Complementary and Alternative Medicine includes a range of therapeutic approaches. The National Center for Complementary and Alternative Medicine (NCCAM), within the National Institutes of Health (NIH), defines these healthcare modalities as “a group of diverse medical and healthcare system, practice, and products that are not generally considered part of conventional medicine although many of these treatments are aimed at promoting health and/or preventing diseases.” [44].

Despite the increasing popularity of CAM use among children in the U.S., few datasets are available for CAM use among pediatric population at a national level. The 2012 NHIS Child Complementary and Alternative (CAL) supplement provides the most comprehensive and current information about CAM use by U.S. children nationwide. In particular, the 2012 CAL supplement collected information about all sample children aged from 4 to 17 years old on their use of 18 CAM therapies: acupuncture, Ayurveda, biofeedback, chelation therapy, chiropractic or osteopathic manipulation, craniosacral therapy, energy healing therapy, hypnosis, massage, naturopathy, traditional healers, movement therapies (Pilates/Trager psychophysical integration/ Feldenkrais), herbal and non-vitamin supplements, vitamins and minerals, homeopathy, special diets, yoga/tai chi/qi gong, and relaxation techniques (meditation/guided imagery/progressive relaxation).

While some questions were asked for participants regarding each one of the CAM therapies, others were asked only for the top three CAM modalities (i.e., herbal remedies, mind-body therapies, and chiropractic care) deemed by the respondents to be most important to the sample child’s medical conditions and health. For example, lifetime prevalence of CAM use was queried with the question: Have you ever used/practiced “a specific CAM modality” (e.g., yoga, meditation, tai chi, or qi going)? Those who answered “yes” were asked with an additional question on CAM use in the past 12 months then were presented with further questions. These questions include: 1) Which health issues do you use CAM for? 2) What are the reasons for using the top 3 CAM modalities? 3) How helpful are the top 3 CAM modalities for the health issues? 4) Where did you (parents of children) get information about mind-body therapies? 5) Who recommend you (parents of children) to use mind-body therapies? and 6) Did you tell your personal healthcare providers about using these CAM therapies?

**Fig. 1:**
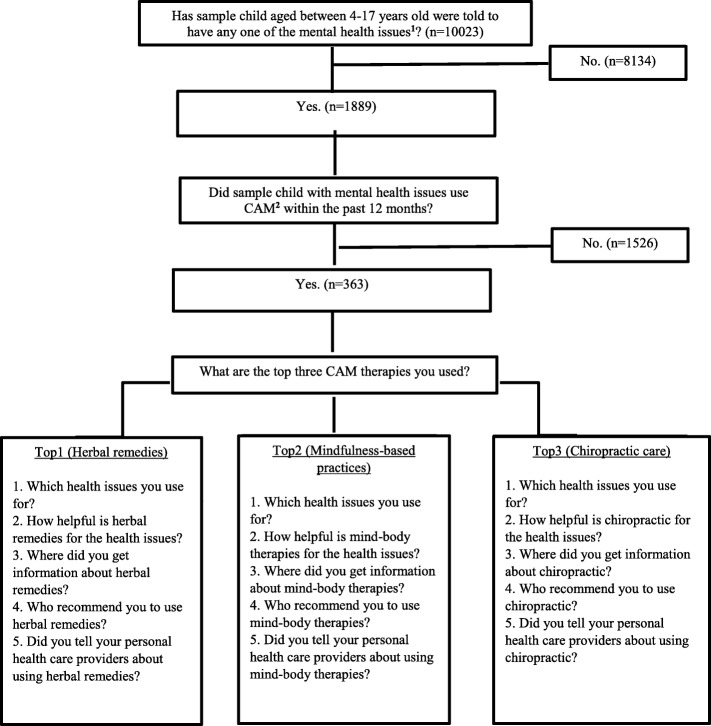
Flow Chart of CAM use among children with mental health issues: 2012 NHIS

A brief description of the questions regarding CAM use among children with mental health issues is depicted in Fig. 1. More information about the detailed questions can be found online [45].

##### Covariates

Based on findings from previous studies, we examined socio-demographics, health insurance status, healthcare-related factors, and several common medical comorbidities (e.g., back/neck/head pain, asthma, allergies, and sleeping problems) to identify the factors that are potentially associated with CAM use among children with mental health issues [2]. Social-demographic factors and health insurance status were primarily derived from the Family Core and Sample Child Core, both of which were categorized based on the earlier NHIS analyses and previous studies [46]. In particular, these factors included age (4–7, 8–11, 12–17 years), gender (boys vs. girls); race (non-Hispanic White vs. black, Hispanic or other race); highest education of either parent (high school graduate or less, some college, and bachelor’s degree or more); annual household income (≤$34,999, $35,000--$74,999, and ≥ $75,000); region of the country (Northeast, Midwest, South, West); health insurance status (yes vs. no). Children’s BMI was classified as underweight, normal weight, and overweight or obesity based on age specified 25th and 85th percentiles of BMI cut-points. BMI was only recorded for children 12 and older in the 2012 NHIS [47].

### Health care-related factors

The Sample Child Core provided data on sample child’s use of and access to traditional medical care. Several question were used in this study to explore if CAM use is significantly associated with the use of and access to traditional medical care use, which include: 1) *Is there a place that [S.C.name] USUALLY goes when [he/she] is sick or you need advice about [his/her] health?* 2) *DURING THE PAST 6 MONTHS, was [S.C. name] prescribed medication or taking prescription medication for difficulties with emotions, concentration, behavior, or being able to get along with others?* 3) *DURING THE PAST 12 MONTHS, was there any time when [S.C. name] needed prescription medicines, but didn’t get it because you couldn’t afford it?* 4) *DURING THE PAST 12 MONTHS, have you seen or talked to a mental health professional such as a psychiatrist, psychologist, psychiatric nurse, or clinical social worker about [S.C. name] health?* 5) *DURING THE PAST 12 MONTHS, was there any time when [S.C. name] needed mental health care or counseling, but didn’t get it because you couldn’t afford it?* Based on the suggestions from previous studies, we collapsed data regarding delayed medical care due to access difficulties (difficulty getting through on phone, couldn’t get an appointment soon enough, wait too long to see a doctor, wasn’t open when you could get there, didn’t have transportation) into a single dichotomous category to explore this possible correlates with CAM use (delay vs. no delay in medical traditional care access) [2].

### Co-morbid medical conditions

To identify the relationship between CAM use and the presence of co-morbid chronic medical conditions among children with mental health conditions, we examined seven co-morbid medical conditions which are potentially associated with CAM use in children with mental health conditions. We placed into three groupings of variables: 1) asthma or respiratory allergies; 2) head/neck/back pain (respondents indicated “headache or migraine” or “neck pain” or “low back pain”); and 3) sleep problems (“excessive sleepiness” or “insomnia”). Among children with mental health conditions, the percentage of CAM use was estimated and then compared between those with and without the co-morbid condition.

### Statistical analyses

We used merged data from the Family Core, Sample Child Core, and the CAL supplement of the 2012 NHIS for data analyses. Weights, Strata, and primary sampling units (PSUs) were used to account for the complex sampling strategy including stratification, clustering, and oversampling of specific populations. Population-based estimates were achieved by using weights calibrated to the 2010 census-based population estimates for age, gender, and ethnicity of the US civilian non-institutionalized population.

Descriptive analysis was performed to describe the prevalence of the four most commonly mentioned mental health issues in children. National weighted estimates are produced for the prevalence of having each of four mental health conditions and the percentage that had any of these conditions. Prevalence (with standard error) was estimated for the overall population and demographic subgroups. Differences across subgroups were compared using Chi-square Wald F-tests.

To determine the most frequently used CAM therapies by children with mental health issues, we first combined all individual CAM modalities into an overarching category of any CAM use in the past 12 months. Then we performed descriptive analysis to identify the top six most commonly used CAM therapies for mental health issues based on their percentages. We also performed descriptive analysis for the series of questions that were asked for the top three specific CAM therapies, including the reasons for using the specific CAM, perceived helpfulness of using CAM for their health issues, the information sources of their CAM use, the referrals for them to use CAM, and whether they disclosed their CAM use to their medical doctors. In all analyses, Ayurveda and chelation therapy were excluded because they had very low prevalence (< 0.1%) precluding meaningful statistical analysis, whereas vitamins and minerals were excluded due to their high prevalence (55%) that suggested their widespread consumption to promote general health over any distinct CAM therapy use.

For analysis of the top three CAM therapies, we combined Yoga/Tai Chi/Qigong, deep breathing, and meditation into one category of mindfulness-based practices for further analysis and investigation. The rationale for doing so is that these techniques are generally associated with relaxation. In addition, they all focus on interactions among the brain, mind, and body with the intent to use the mind to affect physical functioning and promote health. Moreover, deep breathing exercises and meditation are important parts of yoga, tai chi, and qigong. Furthermore, yoga, tai chi, and qigong commonly emphasize the integrative effects of body movement, deep breathing, and mind regulation on health and diseases (e.g., stress, anxiety, and depression) [26, 48].

Chi-square tests with Wald F-statistics were conducted to compare: 1) the percentage of CAM use between children with and without any of these mental health conditions, 2) those with and without the co-morbid medical conditions, and 3) those who answered yes or no to each of six factors related to the use of and access to traditional medical health care among children with mental health conditions.

Multivariable logistic regression models were performed to identify the factors associated with CAM use among children with mental health issues. A backward elimination approach was used to identify potential factors independently associated with CAM use among children with mental health issues. Among the potential factors entered in the initial regression model, we only selected those factors associated with CAM use at a *p*-value of ≤.20 in bivariate analysis. Then, we added sociodemographic factors while adjusting for age, sex, and race/ethnicities. Finally, we adjusted for other sociodemographic factors that may be associated with CAM use, including parents’ education, family income, geographic regions, children’s body weight status (BMI), health insurance status, and co-morbid medical conditions.

For regression analyses, mental health conditions were combined into a single category of having at least one of the four most commonly mentioned mental health conditions. To compare the individual influences that different predictors had on CAM use among children with mental health issues, adjusted odds ratio (AOR) with 95% Confidential Intervals (95% CI) were calculated in line with prior NHIS analysis [46]. Statistical significance for the outcomes in the logistic models was defined as *P* < 0.05.

Data management and statistical analyses were performed using SAS version 9.4 (SAS Institute Inc., Cary, NC). PROC SURVEYMEANS was used to obtain prevalence estimates and standard errors. PROC SURVEYFREQ was used to perform the Wald F-tests. PROC SURVEYLOGISTIC was performed to obtain AOR and 95% CI. Statistical significance for prevalence estimates and standard errors was set as *p*-value < 0.001 based on general considerations for multiple hypothesis testing and the Bonferroni adjustment criterion (*P*-values < 0.01 were considered nearly significant).

All of the prevalence estimates with standard errors, Rao-Scott design-adjusted Chi-square tests (Wald F-tests), and logistic regression analysis was adjusted for the complex survey sampling design that involved sampling weights, adjusting for clustering of respondents, and oversampling of some specific sub-populations.

## Results

Based on the 2012 NHIS survey description, there were 13, 275 children that were interviewed prior to the survey year [49]. Only parents whose children aged 4 to 17 years old (*n* = 10,218) were asked about CAL supplement questions; an additional 195 children were omitted because they did not answer any questions in the CAL supplement, resulting in 10,023 children in this study.

Among the n = 10,023 children in this study, there were 5105 boys (50.9%) and 4918 girls (49.1%). There were 5485 children less than 12 years of age, whereas 4538 children were 12 years old or greater. Among the latter group, 266 (5.9%) children had missing BMI, which was not imputed. Using conditional mode imputation, family income was imputed for 451 of 454 children with missing income, and for 6 of 9 children with missing parent education. Simple mode imputation was used for Health Insurance Status where refused (0.05%) and didn’t know (0.02%) were imputed to “yes” (*n* = 7). For mental health conditions, comorbid conditions and healthcare-related factors, “refused” and “don’t know” are imputed as “no” (< 0.1%), except missing data are imputed as “yes” for the health care access (“Have a place to go” when sick, < 0.1%).

**Fig. 2:**
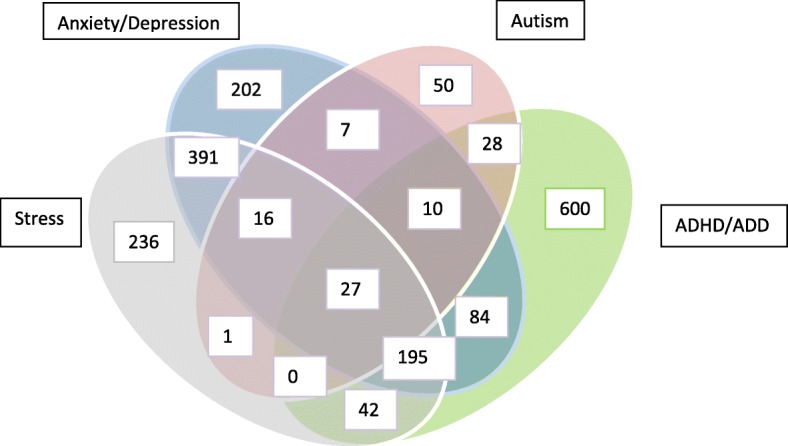
Combinations of mental health conditions for the 1889 children responders in the NHIS CAL supplementary questionnaire with at least one mental health condition

Among all participants, 8134 (81.2%) reported no mental health condition, whereas 1889 (18.85%) reported at least one of the four mental health conditions. A total of 1088 children (10.9%) of the 10,023 reported exactly one condition, 553 (5.5%) reported two conditions, 221 (2.2%) reported three conditions and 27 reported all four conditions (0.3%). The frequencies of these self-reported mental health issues in children are depicted in Fig. 2.

Sample survey weighted estimates, calculated from the 10,023 children who responded to the CAM questionnaire, indicate that 10.1% (standard error [s.e.] 0.4%) of children between the ages of 4 and 17 had a diagnosis of ADHD, 1.3% (s.e. 0.3%) had a diagnosis of autism or autism spectrum disorder, 8.8% (s.e. 0.4%) had a diagnosis of anxiety and/or depression, and 8.9% (s.e. 0.4%) had a diagnosis of childhood stress. Moreover, 18.7% (s.e. 0.5%) of children between the ages of 4 and 17 had at least one diagnosis among these four mental health conditions. The percentages with different mental health conditions varied significantly by gender, age, race, parent’s education and income (*p* < 0.001), but not by BMI, region or health insurance status (Table 1). Exceptions are that the prevalence of ADHD/ADD varied by region and was higher for those with health insurance (10.3%) than those without insurance (5.1%). ADHD/ADD and Autism/ASD was significantly higher in boys than girls. Anxiety/depression in non-Hispanic whites (9.4%) was significantly higher than for other races (7.0%), and childhood stress increased as children aged.

**Table 1:** Percentage of children in demographic subgroups^a^ (standard error) with mental health condition^b^ for the association of the demographic characteristic with the condition

Demographics	N	ADHD/ ADD	Autism/ autism spectrum	Anxiety/ Depression	Childhood Stress	Any Mental Health Condition
Gender						
Boys	5105	14.3 (0.6) ^††^	2.2 (0.3) ^††^	8.7 (0.5)	8.4 (0.5)	21.9 (0.7) ^††^
Girls	4918	5.7 (0.4)	0.4 (0.1)	8.9 (0.5)	9.4 (0.5)	15.4 (0.7)
Age						
4–7	2755	5.1 (0.5) ^††^	1.3 (0.3)	2.7 (0.4) ^††^	2.0 (0.3) ^††^	8.7 (0.7) ^††^
8–11	2730	11.8 (0.8)	1.5 (0.3)	9.8 (0.7)	8.3 (0.6)	19.5 (0.9)
12–17	4538	12.2 (0.6)	1.2 (0.2)	12.2 (0.6)	13.8 (0.7)	24.8 (0.8)
BMI^c^						
Underweight	558	15.1 (1.9)*	0.8 (0.4)	12.4 (1.8)	12.0 (1.6)	27.2 (2.4)
Normal Weight	2237	10.6 (0.8)	1.2 (0.3)	12.1 (0.9)	14.7 (1.0)	23.9 (1.1)
Overweight/Obesity	1743	13.4 (1.1)	1.5 (0.4)	12.2 (1.0)	13.1 (1.0)	25.0 (1.4)
Race						
Non-Hisp. White	7069	10.6 (0.4)*	1.3 (0.2)	9.4 (0.5) ^††^	9.5 (0.5) ^†^	19.7 (0.6) ^††^
Black/Hisp./Other	2954	8.8 (0.7)	1.5 (0.3)	7.0 (0.6)	7.1 (0.6)	15.9 (0.9)
Parent’s Education						
High school or less	3198	10.3 (0.7) ^††^	0.8 (0.2)	8.4 (0.7)*	8.1 (0.7) ^†^	17.9 (0.9) ^††^
Some college	3521	12.2 (0.7)	1.7 (0.3)	10.1 (0.6)	10.5 (0.6)	21.9 (0.8)
Bachelors or higher	3301	8.1 (0.6)	1.4 (0.2)	7.9 (0.6)	7.9 (0.6)	16.3 (0.8)
Income						
≤ $34,999	3584	12.1 (0.8) ^†^	1.1 (0.2)*	10.4 (0.6) ^†^	9.9 (0.7)	22.0 (1.0) ^††^
$35,000–$74,999	3024	9.8 (0.7)	2.0 (0.3)	8.8 (0.7)	9.0 (0.7)	18.1 (0.9)
≥ $75,000	3412	8.8 (0.6)	1.0 (0.2)	7.5 (0.6)	7.9 (0.6)	16.4 (0.8)
Region						
Northeast	1624	8.5 (0.8) ^††^	1.2 (0.3)	8.4 (0.7)	7.7 (0.9)	15.8 (1.0) ^†^
Midwest	1958	11.7 (1.0)	1.3 (0.3)	9.8 (0.8)	9.4 (0.8)	20.3 (1.2)
South	3616	12.0 (0.6)	1.6 (0.3)	8.4 (0.6)	8.6 (0.6)	20.2 (0.8)
West	2825	6.8 (0.7)	1.0 (0.2)	8.9 (0.7)	9.7 (0.8)	16.9 (1.0)
Health Insurance Status						
Yes	9543	10.3 (0.4) ^††^	1.3 (0.1)	8.9 (0.4)	8.9 (0.4)	18.8 (0.5)
No	480	5.1 (1.2)	1.8 (0.8)	7.6 (1.4)	8.6 (1.5)	15.7 (2.1)

^a^ Percentages are weighted for survey design

^b^ Mental health condition within past 12 months

^c^ BMI was recorded only for children 12 years old and above; among those, 266 had missing BMI leaving 4272 children with BMI recorded. *P*-values for survey weighted Wald F-statistic for testing the association of the demographic characteristic and the presence of the mental health condition: < 0.05*, < 0.01^†^, < 0.001^††^

While 19.2% of children with mental health issues had used one or more types of CAM therapies, only 10.1% of children without a mental health condition used CAM within the previous survey year (*p* < 0.001) (Table 2). The top six most frequently used CAM therapies were herbal remedies at 9.1% (s.e. 0.8%), followed by chiropractic at 5.3% (s.e. 0.7%), yoga at 4.7% (s.e. 0.6%), deep breathing at 4.5% (s.e. 0.5%), meditation at 3.7% (s.e. 0.5%), and homeopathy at 2.5% (s.e. 0.5%).

**Table 2:** Percent CAM use (standard error^1^) for top six CAM approaches used among children aged 4–17 without and with common mental health conditions in the NHIS child CAL supplement

Mental Health Condition	Herbs	Chiropractic	Yoga	Deep Breathing	Meditation	Homeopathy	Any CAM Use
ADHD/ADD							
Yes (*n* = 986)	9.0 (1.1) ^††^	3.2 (0.8)	2.4 (0.6)	2.5 (0.6)	1.9 (0.5)	1.3 (0.5)	14.8 (1.5)*
No (*n* = 9037)	4.4 (0.3)	3.5 (0.3)	3.3 (0.2)	2.7 (0.2)	1.9 (0.2)	1.8 (0.2)	11.5 (0.5)
Autism							
Yes (*n* = 139)	8.0 (2.7)	1.2 (0.1)*	5.9 (2.4)	4.7 (2.1)	5.0 (2.3)	3.9 (2.3)	16.8 (4.5)
No (*n* = 9884)	4.8 (0.3)	3.5 (0.3)	3.2 (0.2)	2.7 (0.2)	1.8 (0.2)	1.7 (0.2)	11.7 (0.5)
Anxiety or Depression							
Yes (*n* = 932)	10.4 (1.2) ^††^	6.9 (1.0)^†^	6.8 (1.0) ^††^	6.7 (1.0) ^††^	6.1 (1.0) ^††^	3.4 (0.8)*	23.2 (1.6)^††^
No (*n* = 9091)	4.3 (0.3)	3.2 (0.3)	2.9 (0.2)	2.3 (0.2)	1.5 (0.1)	1.6 (0.2)	10.7 (0.5)
Childhood Stress							
Yes (*n* = 908)	12.0 (1.4) ^††^	8.0 (1.2) ^††^	6.6 (1.0) ^††^	6.4 (0.9) ^††^	5.1 (0.9) ^††^	4.4 (1.0) ^†^	25.7 (1.9) ^††^
No (*n* = 9115)	4.2 (0.3)	3.0 (0.3)	2.9 (0.2)	2.3 (0.2)	1.6 (0.1)	1.5 (0.1)	10.5 (0.5)
Any Mental Health Condition							
Yes (*n* = 1889)	9.1 (0.8) ^††^	5.3 (0.7)^†^	4.7 (0.6) ^†^	4.5 (0.5) ^††^	3.7 (0.5) ^††^	2.5 (0.5)	19.2 (1.2) ^††^
No (*n* = 8134)	3.9 (0.3)	3.1 (0.3)	2.9 (0.2)	2.3 (0.2)	1.5 (0.1)	1.6 (0.2)	10.1 (0.5)

^1^percentages are adjusted for sample weights and standard errors are adjusted for the complex survey design

^2^Survey weighted Wald F-statistic comparing percent CAM use between children with the mental health condition to those without the mental health condition < 0.05*, < 0.01^†^, < 0.001^††^

^3^Any of 17 Cam modalities listed in the methods section

**Fig. 3:**
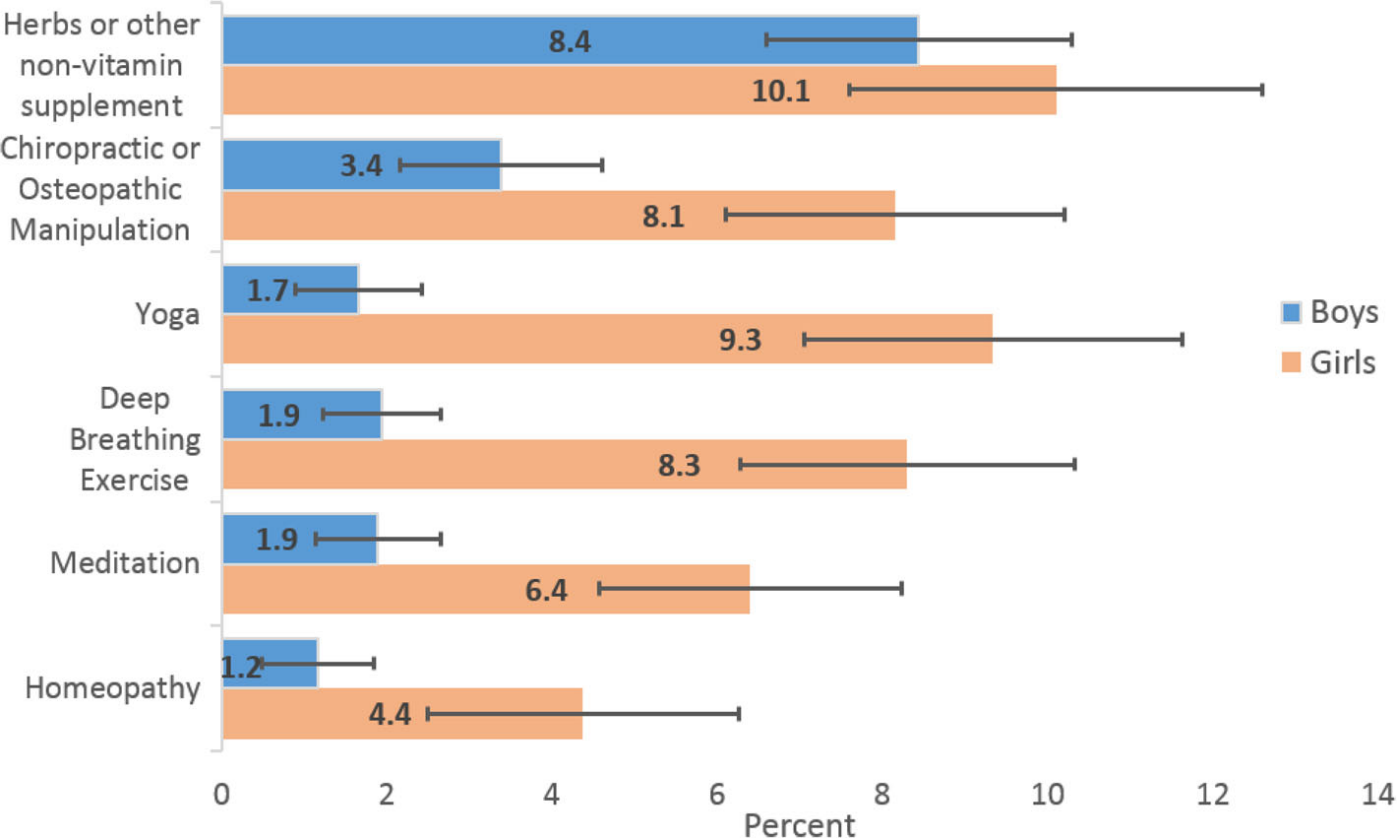
Gender difference in CAM use among children with mental health issues

Autism was not statistically significantly associated with any of the CAM modalities, perhaps due to the small sample size. Otherwise, children with ADHD/ADD used herbs significant more than children without ADHD/ADD; children with Anxiety of Depression used herbs, yoga, deep breathing, meditation and any CAM use more than children without anxiety or depression (*p* < 0.001); and children with childhood stress used herbs, chiropractic, yoga, deep breathing, meditation and any CAM use more than children without childhood stress; and children with any mental health condition used herbs, deep breathing, meditation and any CAM use more than children without any of the four mental health conditions. Additionally, CAM use was higher in girls than in boys, and girls with mental health conditions used CAM statistically significantly higher than girls without mental health conditions for the top six CAM modalities (*p* < .001; Fig. 3).

Prevalence of CAM use was significantly different between those with and without any mental health condition in almost every demographic subgroup examined, with the subgroup of children aged 4–7 years old being a notable exception (Table 3). For example, boys with a mental health condition have 14.3% CAM use versus 8.7%for boys with no mental health conditions. Also, children who have insurance show significant or nearly significant differences between at least one of the four mental health and no mental health conditions in terms of the six most prevalent CAM (19.4% vs. 10.2%, *P* < 0.01).

**Table 3:** Percent CAM use^a^ (standard error) by demographics among children with mental health conditions and without any mental health condition during the past 12 months

Demographics	ADHD/ADD (*n* = 986)	Autism/autism spectrum (*n* = 139)	Anxiety/Depression (*n* = 932)	Childhood Stress (*n* = 908)	Any Mental Health Condition (*n* = 1889)	No Mental Health Conditions (*n* = 8134)	*P*-value^b^
Gender							
Boys	12.8 (1.4)	13.9 (1.1)	18.6 (1.8)	19.4 (2.0)	14.3 (1.4)	8.7 (0.6)	<.001
Girls	20.1 (2.8)	33.1 (5.0)	27.8 (2.2)	31.6 (2.3)	26.5 (2.0)	11.4 (0.7)	<.001
Age							
4–7	10.1 (2.9)	5.7 (2.7)	11.0 (1.8)	11.5(2.4)	8.9 (2.2)	8.2 (0.7)	0.74
8–11	14.9 (2.6)	28.8 (1.9)	24.1 (2.5)	28.6 (2.0)	20.0 (2.1)	8.3 (0.8)	<.001
12–17	16.0 (1.8)	14.6 (1.3)	24.4 (1.7)	25.9 (2.0)	21.1 (1.5)	13.0 (0.9)	<.001
BMI^c^							
Underweight	25.7 (4.0)	33.0 (2.3)	32.0 (4.4)	27.8 (4.7)	27.6 (4.2)	16.3 (2.5)	<.001
Normal Weight	17.1 (2.3)	18.0 (1.1)	29.3 (2.5)	32.1 (2.7)	25.2 (2.2)	14.9 (1.2)	<.001
Overwght/Obese	10.7 (1.7)	7.3 (1.2)	14.8 (2.4)	15.5 (2.7)	13.2 (1.9)	9.2 (1.1)	0.069
Race							
Non-Hisp. White	15.6 (1.5)	18.1 (1.6)	23.9 (1.6)	27.2 (1.8)	20.5 (1.4)	10.8 (0.6)	<.001
Black/Hisp/other	11.8 (2.3)	13.4 (1.0)	20.3 (3.2)	19.8 (3.4)	14.2 (2.0)	8.2 (0.8)	.004
Parent’s Education							
High Sch. or less	10.0 (2.2)	8.2 (0.7)	10.6 (2.1)	12.2 (1.9)	9.3 (1.5)	4.3 (0.6)	.002
Some college	10.8 (1.5)	14.5 (1.0)	20.7 (2.1)	24.7 (2.5)	16.7 (1.6)	9.6 (0.7)	<.001
≥ Bachelor’s	25.4 (2.8)	23.5 (2.7)	36.9 (3.2)	38.3 (3.2)	31.1 (2.4)	15.1 (1.0)	<.001
Income							
≤ $34,999	6.5 (1.2)	12.7 (0.8)	13.3 (1.9)	14.4 (2.0)	9.4 (1.2)	6.0 (0.6)	.013
$35,000–$74,999	13.2 (2.0)	14.5 (2.2)	21.5 (2.7)	28.5 (3.1)	19.1 (2.2)	9.4 (0.8)	<.001
≥ $75,000	25.5 (3.0)	24.3 (2.5)	36.0 (3.2)	34.9 (2.8)	30.2 (2.4)	13.9 (0.9)	<.001
Region							
Northeast	17.7 (4.4)	9.2 (1.3)	19.6 (3.6)	22.0 (3.0)	17.6 (3.1)	9.8 (1.2)	.020
Midwest	15.0 (1.8)	18.5 (1.5)	25.7 (3.0)	24.9 (3.5)	19.6 (2.1)	13.2 (1.3)	.012
South	10.6 (1.7)	19.7 (1.9)	16.1 (1.9)	21.1 (2.2)	14.3 (1.5)	6.4 (0.5)	<.001
West	23.4 (4.5)	13.7 (3.6)	33.3 (3.1)	35.0 (3.5)	28.9 (3.3)	13.0 (1.1)	<.001
Health Insurance Status							
Yes	15.0 (1.3)	15 (1.2)	23.6 (1.4)	26.2 (1.6)	19.4 (1.2)	10.2 (0.5)	<.001
No	3.3 (3.2)	-- (−-)^d^	10.6 (5.4)	13.2 (5.4)	12.5 (3.5)	9.0 (1.7)	0.32

^a^ Any of 17 Cam modalities

^b^ Survey weighted Wald F-statistic comparing percent CAM use between children with any mental health condition to those children with no mental health condition

^c^ BMI was recorded only for children 12 years old and greater

^d^ Unreliable estimates due to there being only six children with Autism/autism spectrum disorder from families without health insurance are not reported

**Fig. 4:**
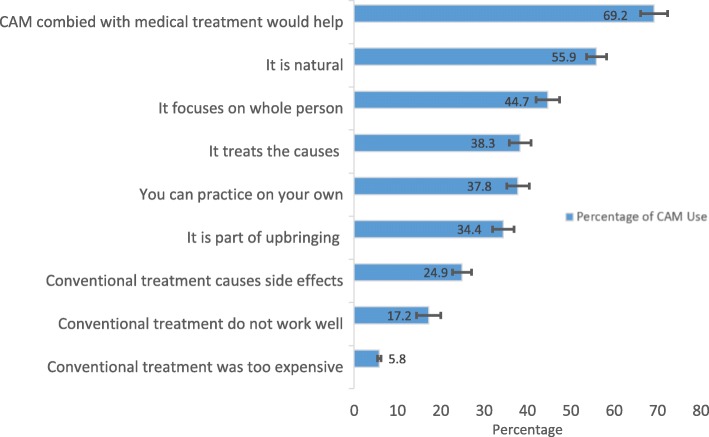
Reasons for using the top three CAM therapies for children with mental health issues

**Fig. 5:**
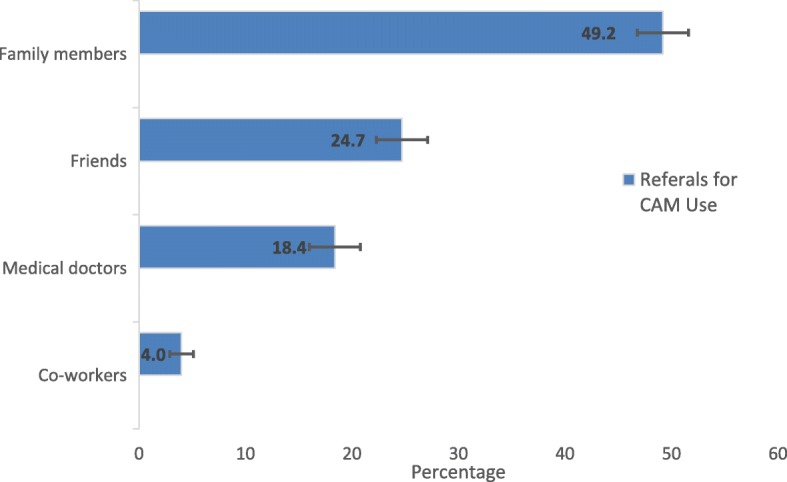
Referrals for using the top three CAM therapies for children with mental health issues

The primary reasons for using any of the top three CAM modalities among children with mental health conditions were: 1) the CAM combined with conventional treatment would help (69.2%), 2) they are natural (55.9%), 3) they focus on the whole person (mind-body-spirit) (44.7%), 4) and they treat the cause and not just the symptoms (38.3%). Only a few pediatric CAM users (5.8%) reported their use of CAM was because conventional medical treatment was too expensive (Fig. 4). Among these CAM users, 49.2% of them were recommended by family members. However, only 18.4% were recommended by medical doctors (Fig. 5). While the majority of CAM users perceived CAM therapies as helpful, only about one-third of them disclosed their CAM use to their medical doctors.

Sample survey weighted estimates, calculated from the 1889 parents of children reporting any mental health condition, showed that 55.6% (s.e. 1.5%) of children between the ages of 4 and 17 reported at least one of the seven comorbid conditions, 32.4% (s.e. 1.4%) reported asthma or respiratory allergy, 25.8% (s.e. 1.4%) reported problems with headaches/migraines, neck pain or low back pain, and 25.9% (s.e., 1.3%) had sleep problems. The prevalence of any CAM use was significantly increased when having at least one comorbid chronic condition compared to when having no comorbid conditions (24.1% vs. 13.0%, respectively, *p* < 0.001; Table 4). Moreover, any CAM use was significantly associated with head/neck/back pain and sleep problems (*P* < 0.001) and nearly significantly associated with asthma and/or respiratory problems (*P* = 0.006).

**Table 4:** Percent CAM use^a^ in the last 12 months (standard error) by comorbid chronic condition among *n* = 1889 children reporting any mental health condition

Comorbid Chronic Condition	N	Percent CAM use (standard error)	*P* Value^b^
Any Condition			<.001
Yes	1067	24.1 (1.6)	
No	822	13.0 (1.4)	
Asthma or respiratory allergy			.006
Yes	635	24.4 (2.3)	
No	1254	16.7 (1.4)	
Head/neck pain/back pain^c^			<.011
Yes	501	26.6 (2.2)	
No	1388	16.6 (1.2)	
Sleep problems^d^			<.001
Yes	483	26.6 (2.5)	
No	1406	16.6 (1.2)	

^a^ Any of 17 Cam modalities

^b^ Survey weighted Wald F-test comparing percent CAM use between children with and without the indicated comorbid chronic condition within the subpopulation of all children with any mental health condition

^c^ “Head” refers to headaches (including migraines); neck pain and low back pain were missing because children under 6 years (*n* = 54) were not asked these two questions

^d^ Sleep problems is defined as excessive sleepiness or insomnia

CAM use among children with mental health issues was not significantly associated with most of the health care related factors (Table 5), which include: 1) having a place to go when sick (19.1% versus 21.2%; *p* = .759), 2) having prescription medications (18.7% versus 19.4%; *p* = .771), 3) having difficulty in affording prescription medications (22.8% versus 19.0%; *p* = .541), and 4) using mental health care and counseling (19.8% versus 18.9%; *p* = .684). However, children whose families reported difficulty affording mental health care and counseling (*n* = 87) reported higher CAM use than those not reporting such financial obstacles (36.9% versus 18.3%; *p* = 0.012).

**Table 5:** Percentage (SE) CAM use^a^ in the last 12 months by health care related factor among *n* = 1889 children reporting any mental health condition

Health care related factor	N	Percent CAM use (standard error)	*P* Value^b^
Health care access (Have a place to go)			.759
Yes	1808	19.1 (1.2)	
No	81	21.2 (6.5)	
Prescription medications			.771
Yes	633	18.7 (2.1)	
No	1256	19.4 (1.4)	
Difficult affording prescription medication			.541
Yes	102	22.8 (5.7)	
No	1787	19.0 (1.2)	
Use mental health care and counselling			.684
Yes	665	19.8 (1.9)	
No	1224	18.9 (1.3)	
Difficult affording mental health care and counseling			.012
Yes	87	36.9 (6.4)	
No	1802	18.3 (1.2)	
Delayed access to traditional medical care^c^			.042
Yes	1629	18.2 (1.2)	
No	260	25.3 (3.2)	

^a^ Any of 17 Cam modalities

^b^ Survey weighted Wald F-test comparing percent CAM use between children with and without the indicated health care factor within the subpopulation of all children with any mental health condition

^c^ Delayed access due to at least one of the following reasons: difficulty getting through on phone; couldn’t get an appointment soon enough; wait too long to see doctor; wasn’t open when you could get there; didn’t have transportation

In the multivariable survey logistic regression models (Table 6), family members with or without health insurance were not associated with any CAM use (*p* = 0.245), whereas higher parental education, higher household income, and living in the Western region of the United States were statistically significant factors associated with CAM use while controlling for age, gender, and race/ethnicities (*p* < 0.01). Moreover, normal weight children had approximately 1.8 times higher odds for any CAM use than overweight or obese children (95% CI: 1.2, 2.5). Among medical comorbidities, children with excessive sleep problems or insomnia had 1.8 (95% CI: 1.2, 2.5) times higher odds of using CAM treatments than children without such difficulties. Additionally, children having headaches, neck pain, or back pain, and having asthma or respiratory problems were statistically significantly and positively associated with CAM use (*P* < 0.05).

**Table 6:** Multivariable Survey logistic regression for any CAM use in the last 12 months among n = 1889 children reporting any mental health condition

Variable	Odds ratio	95% CI	*P* Value
Female (vs male)	2.16	(1.56, 2.99)	<.001
Age (reference: 4–7 years)			
8–11 years	2.18	(1.21, 3.95)	.010
12–17 years	1.14	(0.58, 2.23)	.710
Bmi (reference: overweight/obese)			
Underweight	1.83	(1.00, 3.35)	.051
Normal weight	1.81	(1.14, 2.86)	.012
Non-Hispanic White (vs other)	1.30	(0.86, 1.98)	.216
Parent’s education (reference: HS or less)			
Some college	1.35	(0.86, 2.12)	.186
At least a Bachelor’s degree	2.31	(1.28, 4.15)	.005
Income (reference: ≤$34,999)			
$35,000–$74,999	2.01	(1.29, 3.13)	.002
≥ $75,000	2.84	(1.67, 4.82)	<.001
Region (reference: South)			
Midwest	1.49	(0.83, 2.68)	.182
South	1.07	(0.59, 1.94)	.815
West	2.59	(1.39, 4.81)	.003
Family has health insurance	0.61	(0.27, 1.40)	.245
Asthma or respiratory allergy	1.69	(1.17, 2.45)	.005
Head/neck pain/back pain	1.44	(1.05, 1.98)	.026
Sleep problems	1.76	(1.22, 2.54)	.001

## Discussion

To the best of our knowledge, this is the first study that examines the characteristics of CAM use in a nationally representative sample of U.S. children with mental health issues. More than 2 million (estimated) U.S. children are having one or more of these mental health issues used one or more types of CAM therapies within the previous 12 months. This rate was substantially higher than the percentage of CAM therapy usage among those without a mental health condition. Our finding is consistent with a previous study which examined the use of CAM therapies among youth with mental health concerns using the 2007 NHIS data [2].

According to our analysis, the reasons for why parents of children with mental health issues chose to use CAM therapies primarily are because of CAM’s complementary role to disease management (e.g., CAM combined with tradition care would help) and symptom relief (e.g., CAM treat the causes and not just the symptoms) as well as CAM’s natural and holistic approach (e.g., CAM therapies focus on the whole person: mind, body, and spirit) [50]. These above-mentioned reasons indicate that people use CAM mainly because of CAM’s natural and holistic approach towards promoting health and preventing disease. Given most parents of the pediatric CAM users perceived CAM therapies as helpful for their mental health issues and essential for their overall health, future studies should explore why and how specific CAM therapies are beneficial to pediatric psychiatric care.

Based on our findings, Herbal remedies, mindfulness-based practice, and chiropractic care are the top three mostly used CAM therapies. Herbal remedies (e.g., *Ginkgo biloba*, Ginseng, St. John’s Wort, Echinacea, and probiotics) are the most frequently used CAM therapies for children with mental health issues. This finding is consistent with several previous studies that have touted herbs as effective therapies for anxiety, depression, ADHD, and autism, notwithstanding concerns about safety, drug-herb interaction, and appropriate dosing in children [27, 51–53]. The mechanisms of how certain herbs may serve as promising alternative therapies for mental health issues, mainly because they involve anti-inflammatory, antioxidative, and antiapoptotic activity that may be useful for treating mental health symptoms [54]. Another explanation may be due to the complexity of psychiatric problems; it is possible that regulating one single target does not exert the antipsychotic effect as effectively as targeting multiple systems. Herbal remedies can help to treat mental disorders by various mechanisms of action in different systems, which may help to explain why many people are gradually turning towards herbal remedies for their holistic approach as well as a low level of toxicity [51]. For example, in recent studies that examined why people use St. John’s wort (*Hypericum perforatum*) in the treatment of mild to moderate depression, 90.4% of the participants felt it may be helpful, and it does not have many side-effects that many conventional medications do [24, 55]. Aside from the effects that herbal remedies on promoting mental health concerns are promising, clinical evidence for their therapeutic efficacy in children is still lacking [54]. Therefore, more clinical trials in investigating their mechanism of action is warranted.

Our findings indicate that mindfulness-based therapies (e.g., yoga, tai chi, qi gong, deep breathing, and meditation) are the second most popular CAM modality for mental health issues, which is in line with several previous studies [51, 56, 57]. The reasons of why mindfulness-based therapies are particularly helpful for mental health issues might be because these techniques are generally associated with relaxation responses and their mindfulness-based components focus on enhancing the capacity of the mind to reduce stress-related symptoms, such as anxiety, nervousness, and worries, via down-regulation of the hypothalamic-pituitary-adrenal (HPA) axis and the sympathetic nervous system [32, 48, 58, 59]. In addition, most of the mind-body practices, such as yoga, tai chi, and qigong, have been used as moderate and meditative exercise. These kinds of mind-body exercises are associated with better psychological health, positive mood, and have anxiety-reduction effects because of the integrative effect of slow body movement, deep belly breathing, and meditation on improving health and preventing disease [58–61]. For instance, numerous studies have suggested benefits to using yoga, tai chi, qigong, and meditation as promising therapeutic interventions on reducing stress and thus, contributes positively to balance in life, well-being, and mental health [29–31, 62–66]. Given that mindfulness-based research is currently dominated by medical studies, future research should explore how to make mindfulness-based practices more attractive and age-appropriate for children to practice on a regular basis (e.g., school-based mindfulness interventions).

One surprising finding from our study is that chiropractic care was used as one of the most important CAM therapies for children with mental health issues. Although chiropractic care was reported as one of the most frequently used CAM therapies for many other medical conditions [3, 67, 68], few randomized control trials have reported significant clinical benefits of chiropractic practices among pediatric population, and the effectiveness of chiropractic care in reducing children’s mental health symptoms [69–71]. One possible explanation is that chiropractic care addresses all aspects of a child’s health issues and strives to restore balance. Therefore, it might have helped improve psychological health along the way as a byproduct [72]. Moreover, the high usage of chiropractic care for children with mental health issues may not necessarily be related to its effectiveness in treating mental health issues. Instead, parental choices about chiropractic care are somewhat shaped by other considerations such as chiropractors and parents sharing a similar belief system and parents’ perceptions of chiropractic care as natural and user-friendly therapy [73, 74].

Six of the seven healthcare-related factors, including having health insurance, having a place to go when sick, taking prescribed meditation, having difficulty affording medication, using psychological counseling, and having delayed access to conventional care were not significantly associated with CAM use among children with mental health conditions. Our findings differ from previous studies that have reported CAM use was due to cost-related difficulties in seeking conventional care [2, 46]. In fact, our study found that CAM use among children with mental health issues is not statistically significantly associated with “whether having difficulty in affording prescription medications.” Notably, inability to afford conventional medical treatment was the least commonly mentioned reason for CAM use in our study. This result differs from several previous studies that found CAM use is associated with higher medical care utilization [2, 46, 75]. In fact, CAM may be able to serve as an essential modality in reducing health care cost, especially when more patients are using CAM than conventional therapy for their mental health issues (e.g., anxiety and depression) [76–79].

While many CAM therapies have been used as promising therapeutic interventions for mental health conditions, it is concerning that the majority of the parents of pediatric CAM users reported that they learned about CAM from a family member or friend rather than from a physician. Based on our findings, surprisingly, only 18.4% of CAM users reported that a medical doctor recommended CAM use, demonstrating a gap in communication between pediatric psychiatrists and patients about CAM use. These results, from one side, may indicate that parents are the gatekeepers of mental health care for their children [46, 80]. From the other side, it also raises concerns about why doctors are reluctant to communicate or recommend CAM therapies to their pediatric patients, especially when more children are using CAM [39].

Given these circumstances, it may be beneficial to provide CAM-related education and training opportunities for pediatric psychiatrists to understand better why more people are using CAM and how CAM may help to improve health and prevent disease [81–83]. In addition, opening the dialogue surrounding CAM use during routine psychiatric care could present a viable solution to addressing the increasing medical challenges---most notably, the increasing rates of mental health disorders in children [84]. Moreover, increasing CAM education for pediatric psychiatrists will allow them to answer patients’ questions as well as provide informative instruction to ensure that the patients get reliable, accurate, and consistent information. Furthermore, raising the conversation will also uplift and support their patients, making them feel empowered to seek alternative, natural, and holistic methods for complicated medical conditions.

Our results indicating the association between the demographic factors and the CAM use are consistent with most of the previous studies [2, 3]. In particular, CAM use among children with mental health issues is significantly related to being female, aged 8 to 11 years old, having healthy body weight, living in the western geographic region of the U.S., and having one or more co-morbid medical conditions. The higher rates of CAM use in children whose parents have higher education and higher family income than their counterparts indicate that there may be disparities for children to access these promising therapies. Thus, the built-up evidence from this study and the literature may convince policymakers to take steps to promote more equitable access to natural and holistic CAM therapies, such as Herbal remedies and mindfulness-based practices, may be beneficial for children from disadvantaged socioeconomic background whose parents may have a low educational level.

### Limitations

Our study has limitations. First, the survey data is prone to recall bias. Mental health issues are based on self-reporting which may not meet standard clinical definitions or testing. Thus, the study may not have captured children with undiagnosed mental health problems. Second, our study population focused on children with mental health problems, however, children did not respond directly to their use of CAM therapies. Therefore, there may be inconsistencies between adult respondents and their children’s actual use of CAM therapies [85]. Lastly, the NHIS was a cross-sectional survey. It is not possible to conclusively determine why children use CAM for their mental health issues, although some questions were asked to help to answer part of this concern.

## Conclusions

Approximately 10 million children with mental health issues are using CAM therapies based on their parents’ report. The main reason why the parents chose CAM for their children was because of their desire for a more natural and holistic healthcare approach. Given that most CAM users’ parents perceived CAM therapies as helpful, future studies should investigate the unique contributions of CAM in pediatric psychiatric care. Because a low percentage of CAM use was recommended by medical doctors, indicating that educational interventions of equipping medical professionals with CAM knowledge and experience will be conducive to more effective patient-pediatrician communication in terms of the CAM use. Parents with higher education and family income compared to those with lower education and family income were more likely to have their children use CAM therapies, therefore, effective strategies are needed to reduce economic disparities in accessing CAM therapies.

## Appendix

### Conditional mode imputations for income and education

There were nine respondents with missing (Family) education and 454 respondents with missing (family) income. Because education and income are highly related, each are imputed by their mode after stratifying on the other one. According to this imputation scheme, those with low education (High school or less) had low income (less $34999) imputed, and vice versa; those with mid education (some college) had mid income ($35,000–$74,900) imputed, and vice versa; those with high education (Bachelor’s or higher) had high income (over $75000) imputed, and vice versa.

- Family whose education is low have frequencies of low, mid and high incomes of 60%, 27% and 8%, respectively.
- Family whose education is college have frequencies of low, mid and high incomes of 35%, 36% and 25%, respectively.
- Family whose education is > = Bachelor degree have frequencies of low, mid and high incomes of 9%, 22% and 63%, respectively.
- Family whose income is $0–$34,999 have frequencies of low, mid and high education of 65%, 35% and 9%, respectively
- Family whose income is $35,000–$74,999 have frequencies of low, mid and high education of 30%, 44% and 26%, respectively
- Family whose income is over $75,000 have frequencies of low, mid and high education of 8%, 27% and 64%, respectively.

Among the 454 respondents with missing income, 179 had partial income information. Note that their incomes between $0–$49,999, over $50,000, or $50,000–$99,999 do not belong to low, mid or high income groups, so they are among those with imputed income according to their education level. After imputation, three children remain with missing values for both income and education.

## Abbreviations

ADHD: Attention-deficient hyperactivity disorder
CAL: Child complementary and alternative medicine supplement
CAM: Complementary and alternative medicine
NHIS: National Health Interview Survey
